# The effect of preoperative dexamethasone on pain 1 year after lumbar disc surgery: a follow-up study

**DOI:** 10.1186/s12871-016-0277-z

**Published:** 2016-11-16

**Authors:** Rikke Vibeke Nielsen, Jonna Fomsgaard, Ole Mathiesen, Jørgen Berg Dahl

**Affiliations:** 1 Department of Neuroanaesthesiology, Rigshospitalet, Glostrup University Hospital, Nordre Ringvej 57, 2600 Glostrup, Denmark; 2 Department of Neuroanaesthesiology, Rigshospitalet, Glostrup University Hospital, Glostrup, Denmark; 3 Department of Anaesthesiology, University Hospital Zealand, Køge Hospital, Køge, Denmark; 4 Department of Anaesthesiology, Bispebjerg Hospital, Copenhagen University Hospital, København, Denmark

**Keywords:** Dexamethasone, Glucocorticoids, Persistent postoperative pain, Preemptive medicine, Spine surgery

## Abstract

**Background:**

It has been hypothesized that dexamethasone can inhibit persistent postoperative pain, but data on humans is lacking and results from animal studies are conflicting. We explored the effect of 16 mg dexamethasone IV administered preoperatively on persistent pain 1 year after lumbar discectomy.

**Methods:**

This is a prospective 1-year follow-up on a single-centre, randomized, and blinded trial exploring the analgesic effect of 16 mg IV dexamethasone or placebo after lumbar discectomy. One year follow-up was a written questionnaire including back and leg pain (VAS 0–100 mm), Short Form 36 survey (SF-36), EuroQol 5D (EQ-5D), OSWESTRY Low Back Pain Questionnaire, duration of sick leave, working capability, contentment with surgical result.

**Results:**

Response rate was 71% (55 patients) in the dexamethasone group, 58% (44 patients) in the placebo group. Leg pain (VAS) was significantly lower in the placebo group compared to the dexamethasone group: 17 (95% CI 10–26) vs 26 (95% CI 19–33) mm, respectively (mean difference 9 mm (95% CI −1 to 0), (*P* = 0.03). No difference regarding back pain. The placebo group reported significantly more improvement of leg pain and were significantly more satisfied with the surgical result. Patients in the dexamethasone group reported significantly higher pain levels in EQ-5D- and Oswestry questionnaires. No difference in the SF-36 survey or daily analgesic consumption.

**Conclusions:**

We found significantly higher pain levels in the dexamethasone group compared to placebo 1 year after lumbar disc surgery.

**Trial registration:**

Clinicaltrials.gov (NCT01953978). Registered 26 Sep 2013.

## Background

An increasing number of studies have explored the role of glucocorticoids as a potential adjuvant in analgesic regimens for acute postoperative pain management [1, 2]. Reviews and metaanalyses’ indicate that dexamethasone in doses of 0.1–0.2 mg/kg provides an opioid-sparing effect and lower pain scores after surgery [1, 2]. Moreover, it is speculated that perioperative administration of glucocorticoids may prevent persistent postoperative pain but follow-up periods have been relatively short and insufficient to detect possible long term beneficial or harmful effects [1–4].

The mechanisms that influence the transition from acute, adaptive postoperative pain to persistent, maladaptive pain are uncertain, but nerve injury and ongoing inflammation play important roles [5]. Peripheral- and central sensitizations are fundamental mechanisms of the development of pain chronicity in the postoperative period. Proinflammatory cytokines secreted at or near the site of a nerve injury are involved in the development and maintenance of central sensitization and neuropathic pain [6, 7]. Glucocorticoids suppress proinflammatory cytokines and induce expression of anti-inflammatory cytokines [7, 8]. By inhibiting the release of prostaglandins, and production of proinflammatory cytokines, excitatory amino acids, and growth factors in animal models, glucocorticoids prevent the development of neuropathic pain [9, 10].

Persistent postsurgical pain is a serious and widely unnoticed clinical problem, difficult to treat and often permanent [5, 11, 12]. The level of acute postoperative pain is associated with the risk of developing persistent pain [13–15]. However, knowledge of the impact of aggressive, early therapy for persistent, postoperative pain is lacking [12, 16]. Therefore, we aimed to explore the pre-emptive effect of 16 mg dexamethasone IV administered preoperatively on persistent postoperative pain 1 year after lumbar disc surgery.

## Methods

This is a prospective 1-year follow up trial on a single-centre, prospective, randomized, and blinded trial exploring the analgesic effect of 16 mg dexamethasone IV on acute postoperative pain after lumbar disc surgery as the main outcome [17]. The trial was approved by the Regional Research Ethics Committee and the Danish Data Protection Agency and registered at clinicaltrials.gov (NCT01953978).

The trial was performed at Glostrup Hospital, Copenhagen University Hospital, Glostrup, Denmark during the period December 7, 2012 to December 7, 2014, and was monitored by the Copenhagen University Hospital Good Clinical Practice Unit. The follow up period was from December 1, 2013 to September 20, 2015. The study fulfilled the guidelines for Good Clinical Practice and the Helsinki Declarations. The protocol, design and reporting of the study complied with the Standard Protocol Items, Recommendations for Interventional Trials (SPIRIT) statement [18], and the Consolidated Standards of Reporting Clinical Trials statement (CONSORT) [19]. The SF-36 version 1 (freeware) was used for the 1-year follow-up. All patients gave written informed consent before participating in the trial.

### In- and exclusion criteria

One hundred-sixty patients undergoing one- or two-level primary lumbar discectomy during general anaesthesia were approached for inclusion in the original trial [17]. Inclusion criteria were age 18–85 years, American Society of Anaesthesiologists (ASA) physical status classification of I to III, and body mass index between 18 and 40 kg/m2. Exclusion criteria were inability to cooperate, inability to speak or understand Danish, previous spine surgery, pregnancy, allergy to drugs applied in the trial, daily use of systemic steroids or strong opioids (morphine, oxycodone, methadone, fentanyl, or ketobemidone), and alcohol or drug abuse (see also [17]).

### Randomization and blinding

In the original trial, patients were randomly assigned to one of two groups: Oral paracetamol + oral ibuprofen + IV dexamethasone 16 mg; or oral paracetamol + oral ibuprofen + IV placebo. Randomization was performed by the pharmacy according to a computer-generated block randomization list (each block containing 10 numbers), in a 1:1 ratio. Study medication was pre-packed by the pharmacy in consecutively numbered boxes according to the computer generated randomization list, containing identical 1 ml ampules of either dexamethasone 4 mg/ml (Dexamethasone®, GALEN pharma GmbH, Germany), or isotonic sodium chloride 9 mg/ml. The intervention was blinded to patients, investigators, surgeons, and clinical personnel (for further information, please see [17]).

The randomization code was disclosed until a three months follow-up period had ended, exclusion of patients was decided, and statistical handling of the data was completed, but prior to the 1-year follow-up.

The primary outcome of the original trial was pain during mobilization 2 to 24 h postoperatively calculated as a ‘weighted average level’ area under the curve (AUC) (in mm) [17].

Secondary outcomes included pain at rest (AUC, 2–24 h), total morphine consumption 0–24 h postoperatively, and 3- and 12-months follow-up (for further information, see [17]).

### One-year follow-up

The 1-year follow-up was performed by a written questionnaire. The questionnaire was used with permission from Centre for Rheumatology and Spine Diseases, Glostrup University Hospital, Denmark, and the Danish Spine Database. This questionnaire was developed to construct a national database that systematically collects data on patients undergoing spine surgery. The questionnaire consists of demographic data, back and leg pain (VAS 0–100 mm), duration of sick leave, working capability and contentment with the results of the operation. Further it contains the following questionnaires: Short form 36 survey (SF-36), EuroQol 5D (EQ-5D) and OSWESTRY Low Back Pain Questionnaire.

If patients had not returned the questionnaire after three weeks, they received one written reminder.

### Statistical methods

We randomized 160 subjects in the original study, and data on acute pain were analysed in 77 and 76 patients in the dexamethasone and placebo groups, respectively [17].

For the present data, we used SPSS version 22.0 for Windows (SPSS, Chicago, Illinois) for statistical analysis. Variables were tested for normal distribution with the Kolmogorov-Smirnov test and visual inspection. Data that followed normal distribution were compared using the independent samples t test. The Mann–Whitney *U*-test was used for data that were not normally distributed. Categorical data were analyzed using the *x* ^2^ test or Fisher’s exact test if any cells had expected counts less than five. Data are presented as mean (SD), mean (95% confidence interval), median (lower and upper quartiles) or frequencies (95% confidence interval), as appropriate. A sensitivity analysis was performed for missing data on the VAS pain scores with multiple, average, best case and worst case imputation.

OSWESTRY Index Score is calculated using the following method: Questions are rated on a scale of 0–5 with zero recorded as no disability and five recorded as maximum disability. The scores from all questions are summed, and then multiplied by two to attain an index (range 0–100). Zero is then no disability and 100 is maximum disability.

The SF-36 questionnaire is summarized as two sets of scores: A profile of eight section scores, and two summary scores, one for the physical component (PCS) and one for the mental component (MCS) summary scores. Scores are recalculated to normal zero-to-100 scores for the eight scales and all item scores are oriented so that high scores correspond to better health. All scores are calculated according to the formula described by McDowell [20]. Using norm-based scoring (NBS) for the summary scores, each scale is scored to have the same mean (50) and standard deviation (10) as in the general Swedish population, provided by SF-36 organization. Anytime a scale score is below 50, health status is below average relative to the general Swedish population.

The nature of the hypothesis testing was 2-tailed. P values of less than 0.05 were considered statistically significant. As we consider the results of this 1-year-follow-up trial exploratory, outcomes were not statistically corrected for mass significance. The primary investigator carried out statistical analyses.

## Results

**Fig. 1:**
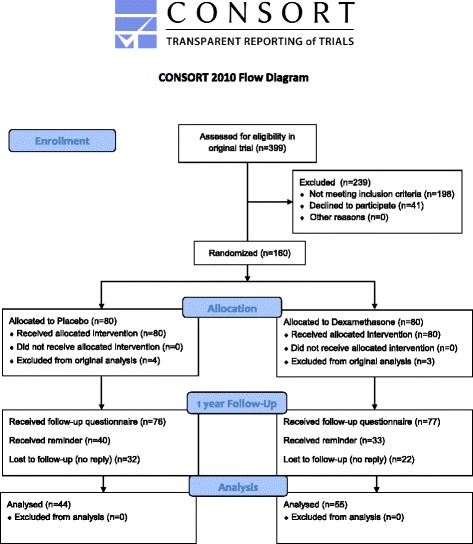
CONSORT flowchart of trial

All 153 patients included in the original data analysis, 77 and 76 subjects in the dexamethasone and placebo groups, respectively, received a follow-up questionnaire 1 year postoperatively. In the dexamethasone group 55 patients (71%) replied, and in the placebo group 44 patients replied (58%), resulting in an overall response rate of 65% (Fig. 1). Patient characteristics (age, height, weight, preoperative pain, postoperative pain 2–24 h and morphine consumption 0–24 h) for responders and non-responders were compared with no significant differences between groups.

### Spine unit questionnaire

**Fig. 2:**
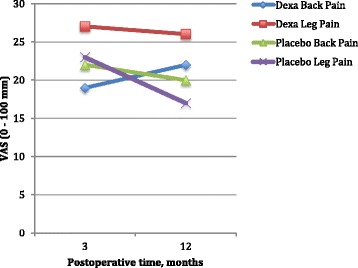
Postoperative back- and leg pain (VAS). Leg pain 12 months postoperatively was significantly less in the placebo group compared to the dexamethasone group: 17 (95% CI 10–26) vs 26 (95% CI 19–33) mm, respectively, with a mean difference of 9 mm (95% CI −1 to 0), *P* = 0.033. There were no other significant differences between groups

For VAS back pain levels at one year postoperatively, there was no significant difference between groups: 22 (95% CI 16–28) vs 20 (95% CI 14–28) mm in the dexamethasone and placebo groups, respectively (mean difference 2 mm (95% CI −1 to 0), *P* = 0.47 (Fig. 2)). Leg VAS pain levels was significantly lower in the placebo group compared to the dexamethasone group: 17 (95% CI 10–26) vs 26 (95% CI 19–33) mm, respectively (mean difference 9 mm (95% CI −1 to 0), (*P* = 0.03) (Fig. 2).

Further, when asked how patients evaluated their leg pain today compared to preoperatively, the placebo group reported a significantly higher degree of improvement of their leg pain, compared to the dexamethasone group: 1 (0 – 5) vs 2 (0 – 5), respectively, (*P* = 0.04) (Table 1). Table 2 summarizes multiple, average, best case and worst case imputation for missing data on pain scores.

**Table 1:** Follow up 12 months postoperatively

	Dexamethasone	Placebo	*P*-value
Patient characteristics			
Number of patients, n	55	44	
Response rate, %	71	58	
Time from operation to follow up, days	364 (12)	366 (11)	0.31
Height, cm	176 (8)	177 (10)	0.41
Weight, kg	79 (14)	83 (14)	0.21
Sex, f/m	27/28	15/29	
Outcome			
Back pain now compared to preoperatively	2 (1 – 3)	2 (1 – 2)	0.19
Leg pain now compared to preoperatively	2 (1 – 3)	1 (1 – 2)	0.04
VAS back pain, 0–100 mm	22 (16–28)	20 (14–28)	0.47
VAS leg pain, 0–100 mm	26 (19–33)	17 (10–26)	0.03
Uses analgesics 12 months postoperatively, %			0.84
Daily	16 (7 – 27)	16 (7 – 27)	
As needed	31 (20 – 45)	36 (23 – 52)	
Currently on sick leave because of back problem, %	13 (4 – 22)	5 (0 – 11)	0.17
Sickleave < 3 months postoperatively, %	79 (58 – 81)	84 (70 – 95)	0.21
Transferred to less demanding job postoperatively, %	15 (4 – 27)	19 (8 – 32)	0.59
Working %	79 (58 – 81)	84 (61 – 87)	0.89
Sports active, %	51 (38 – 64)	68 (48 – 77)	0.09
Walk distance more than 1000 m, %	80 (69 – 89)	91 (82 – 98)	0.21
Satisfaction with surgical result, % (yes/no/unsure)			0.02
Satisfied	66 (51 – 79)	84 (73 – 93)	
Unsatisfied	4 (0 – 9)	7 (0 – 14)	
Unsure	30 (19 – 43)	9 (0 – 18)	

Data are mean (SD), mean (95% CI), median (lower and upper quartiles) or frequencies (95% CI). Data were analysed using the students t-test, Mann–Whitney *U*-test or the *χ*2 test

**Table 2:** Data imputation of pain scores

	Dexa VAS Back	Placebo VAS Back	*P*-value	Dexa VAS Leg	Placebo VAS Leg	*P*-value
Responders only	22 (16–28)	20 (14–28)	0.47	26 (19–33)	17 (10–26)	0.03
Multiple imputation	22 (20–24)	20 (18–22)	0.20	24 (22–27)	17 (16–19)	<0.001
Average imputation	22 (18–27)	20 (17–24)	0.53	25 (20–30)	17 (13–21)	0.03
Best case imputation	11 (7–16)	11 (7–16)	1.00	10 (5–15)	10 (5–15)	1.00
Worst case imputation	44 (36–53)	44 (37–51)	1.00	47 (38–56)	48 (39–56)	0.81

Data are mean (95% CI)

A post hoc analysis of preoperative VAS pain scores and VAS pain during the trial period (weighted average AUC (wAUC) 2–24 h), showed that results from the subgroup of patients that completed the 1-year follow-up were similar to the results from the total study population. Only pain during mobilization was significantly different between groups: Dexamethasone compared to placebo, 33 (22) vs 43 (18) mm (wAUC 2–24 h) with a mean difference of 10 mm (95% CI 3 to 16), (*P* = 0.01) in the original total study population and 32 (20) vs 41 (20) mm with a mean difference of 11 mm (95% CI 2 to 18), (*P* = 0.02) in the subgroup of one-year follow-up responders [17].

**Fig. 3:**
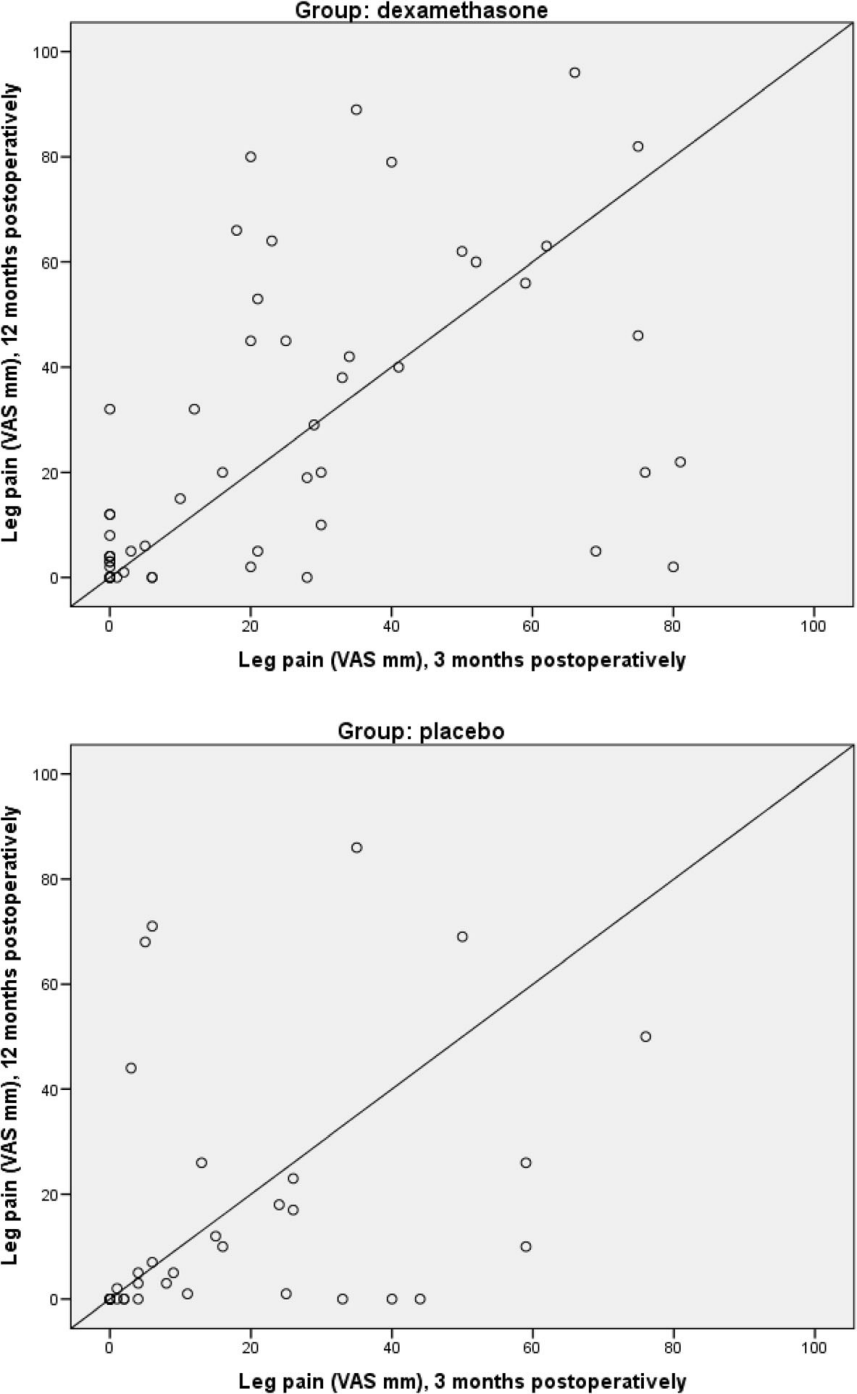
Scatterplot of leg pain (VAS mm) 3- and 12 months postoperatively

The relation between leg pain levels 3- and 12 months postoperatively are summarized in scatter plots (Fig. 3). The frequency of patients having leg pain (VAS) > 30 mm both 3- and 12 months postoperatively was 23% and 8% in the dexamethasone group and placebo group, respectively (*P* = 0.07).

Daily use of analgesics at 1 year postoperatively was not different between groups, with 16% (95% CI 7–27) (*P* = 0.84) having a daily use of analgesics in both groups (Table 1). Preoperatively, the daily use of analgesics was, 64% (95% CI 51–74) in the dexamethasone group and 59% (95% CI 50–69) in the placebo group, including only the patients that completed the 1-year follow up.

Working ability was not significantly different between groups (Table 1). Satisfaction with the surgical result was significantly lower in the dexamethasone group as compared to the placebo group (*P* = 0.02) (see Table 1).

### Short form 36 survey (SF-36)

The SF-36 section scores are summarized in Table 3. Scores were generally lower in the dexamethasone group indicating poorer health, especially regarding physical health, although there were no significant differences between groups. The physical (PCS) component summary score was mean 46 and 50 in the dexamethasone group and placebo group, respectively, *P* = 0.69. The mental (MCS) component summary score was mean 52 and 54 in the dexamethasone group and placebo group, respectively, *P* = 0.49.

**Table 3:** SF-36, eight section scores

	Dexamethasone	Placebo	*P*-value
Physical health			
Physical function	75 (50 – 75)	75 (50 – 100)	0.10
Role physical	75 (0 – 100)	100 (50 – 100)	0.25
Bodily pain	76 (46 – 94)	82 (47 – 100)	0.12
General health	68 (43 – 80)	80 (55 – 93)	0.15
Mental health			
Vitality	60 (40 – 80)	70 (50 – 80)	0.21
Social function	100 (72 – 100)	100 (50 – 100)	0.89
Role emotional	100 (50 – 100)	100 (50 – 100)	0.41
Mental health	70 (58 – 90)	80 (70 – 90)	0.22

Data are median with lower and upper quartiles. Data were analysed using the Mann–Whitney *U*-test. Zero is equivalent to maximum disability and a score of 100 is equivalent to no disability

### EuroQol 5D (EQ-5D)

**Fig. 4:**
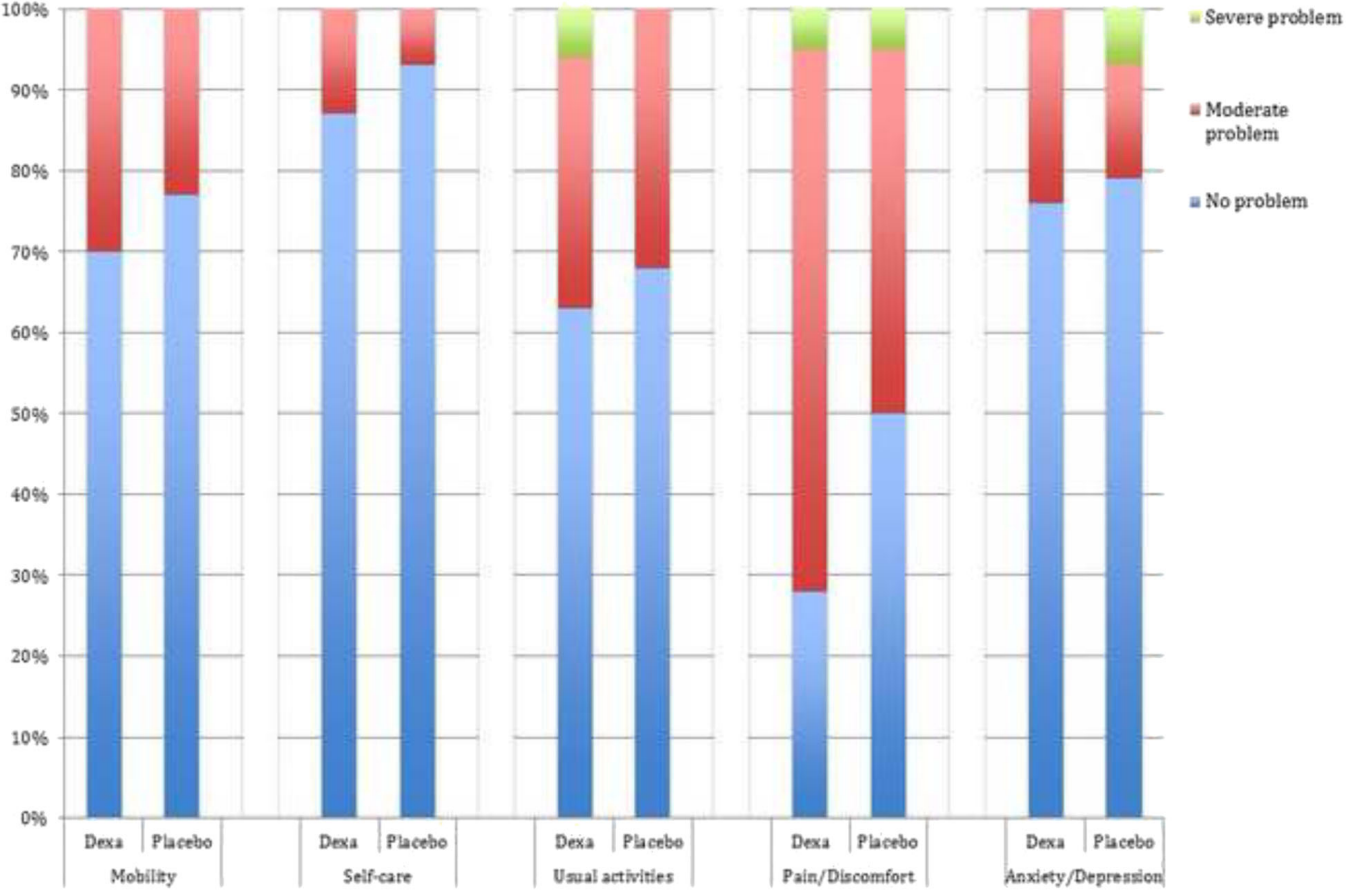
EuroQol. Significant difference in severity of Pain/Discomfort with 28/67/5% in the dexamethasone group reporting no pain/moderate pain/severe pain respectively and in the placebo group 50/45/5% reported no pain/moderate pain/severe pain respectively, *P* = 0.034

Results from the EQ-5D questionnaire are summarized in Fig. 3. There was a significant difference in severity of Pain/Discomfort between groups, with 28/67/5% in the dexamethasone group reporting no/moderate/severe pain, respectively, and 50/45/5% in the placebo group reporting no/moderate/severe pain, respectively, (*P* = 0.03) (Fig. 4).

There was no significant difference between groups in self-reported health (VAS scale) with medians of 80 (4–100) vs 80 (14–100) mm in the dexamethasone and placebo groups, respectively, (*P* = 0.26).

### OSWESTRY low back pain questionnaire

Results from OSWESTRY Low Back Pain Questionnaire are summarized as an Oswestry Index Score 0–100, with 0–20 being minimal disability and 81–100 being patients who are bed-bound. There was no significant difference between groups: Dexamethasone median 14 (0–60) vs placebo median 8 (0–56), (*P* = 0.09), categorizing both groups with minimal disability. However, in the questionnaire, there was a significant difference regarding pain with more severe pain scores in the dexamethasone group: Median 1 (0–5) vs placebo median 1 (0–3), (*P* = 0.01).

## Discussion

In this prospective follow-up on a single-centre, prospective, randomized, blinded trial exploring the analgesic effect of 16 mg dexamethasone IV on acute postoperative pain after lumbar disc surgery, we found significantly higher chronic pain levels in the dexamethasone group at one-year postoperatively. Leg pain was significantly higher in the dexamethasone group compared to the placebo group, measured on two different parameters, as in both the EQ-5D and OSWESTRY surveys, questions on pain/discomfort revealed higher scores in the dexamethasone group.

Further, satisfaction with the surgical result was significantly reduced in the dexamethasone group compared to the placebo group.

It has been suggested that the potent immunomodulatory benefits of dexamethasone could possibly inhibit the development of chronic pain [1, 21]. Few clinical trials have investigated this effect of corticosteroids in postoperative patients [4, 22–25]. Only one trial, testing a bolus and 4 days of intravenous infusion of hydrocortisone, found a significant positive impact on chronic pain 6 months postoperatively [24]. Despite a significant analgesic effect of dexamethasone on acute pain during mobilization 2–24 h postoperatively for both the total study population and in the subgroup of 1-year follow-up responders (post hoc analysis), we were not able to demonstrate any inhibitory effects of dexamethasone on chronic pain 1 year postoperatively.

In contrast we found increased pain levels in the dexamethasone group, specifically leg pain, indicating increased levels of neuropathic pain. In our original trial, 16% (95% CI 7–26) versus 8% (95% CI 0–17) reported new weakness and/or paralysis of the legs affecting the ability to walk in the dexamethasone and placebo groups, respectively, 3 months postoperatively (*P* = 0.20) [17]. It is uncertain whether this result is related to our findings of leg pain 1 year postoperatively, but we did find a significantly higher number of patients in the dexamethasone group with leg pain > 30 mm both 3- and 12 months postoperatively.

Recent experimental trials in rats have demonstrated that glucocorticoid receptors in the spinal cord are up-regulated after constriction nerve injury, and imply that glucocorticoids can worsen neuropathic pain behavior [26–29]. These results imply an important function of neuronal glucocorticoid receptors in the mechanisms of neuropathic pain behaviors in rats and suggest a possible role for glucocorticoid receptor antagonists in the treatment of neuropathic pain. Other studies have found that by inhibiting the release of prostaglandins, and production of proinflammatory cytokines, excitatory amino acids, and growth factors in animal models, glucocorticoids prevented the development of neuropathic pain behavior [9, 10]. Therefore, at present, results on the effect of glucocorticoids after nerve injury are conflicting. Even when adjusting for non-responders by data imputation we found no preventive effect of dexamethasone on persistent pain. When performing multiple- and average imputation we found similar results to those of the responders with higher levels of leg pain in the dexamethasone group. When performing best case- and worst case imputations we found no differences between groups.

Despite significantly higher pain levels in the dexamethasone group we did not find significant differences in ability to work, length of sick leave, disability or self-reported health between groups. However, for both the dexamethasone and placebo groups, pain levels were low in the Spine Unit Questionnaire and EQ-5D, and low to moderate in the Oswestry Index Score for pain.

Elevated blood glucose levels are a potential concern of glucocorticoids, but the hyperglycemic responses measured in current trials are modest at most. Several large reviews have investigated this risk; and so far, there is no solid evidence that a single dose of glucocorticoids is associated with hyperglycemic responses affecting postoperative morbidity [1, 2, 30, 31]. Based on this, we did not measure blood glucose in our study.

A major limitation of this 12 months follow-up study is the response rate of 65%, rendering data with low strength to draw final conclusions on dexamethasone’s role in preventing persistent postoperative pain. We do not know the status of patients that did not respond. If patients with high pain levels are more motivated to respond this could potentially lead to a false positive result (type 1 error). However, this should be similar in both groups. We found no differences in patient characteristics between responders and non-responders. Further our imputations of missing data did not contribute with new indications that dexamethasone can prevent persistent postoperative pain. Our study contains exploratory secondary outcomes from an original trial. Thus, the sample size calculation was not performed with regards to these secondary outcomes. Many of the questions in the questionnaires regard the same topic and we would therefore have to do multiple corrections introducing a greater risk of ignoring a relationship that is real and performing a type 2 error. As we consider these outcomes hypothesis generating, we chose not to correct for multiple comparisons and interpret the results with respect to the increased risk of type 1 errors.

Further this study suffers from the well-known weaknesses of written questionnaires: Suboptimal response rates, a level of subjectivity, recall bias, interpretation of questions, and researcher imposition. The strength of the study is that very few investigators were involved in the trial, leading to few protocol violations, and very few original data missing.

Future studies on possible effects and safety aspects of dexamethasone on sustained postoperative pain are warranted. We consider our results on back pain incidence after lumbar back surgery as a significant negative finding in this study. The exact dose of glucocorticoids at which potential harm outweighs benefit is still unknown.

## Conclusions

In conclusion, we found no inhibitory effect of dexamethasone on chronic pain 1 year after lumbar disc surgery. In contrast we observed significantly higher pain levels in the dexamethasone group compared to placebo, which may be of concern. Our results may be considered as hypothesis generating for future properly sized studies investigating long-term effects of perioperative glucocorticoids on human postoperative pain.

## Abbreviations

ASA: American Society of Anaesthesiologists
AUC: Area under the curve
CI: Conficence interval
EQ-5D: EuroQol 5D
MCS: mental component summary score
NBS: Norm-based scoring
PCS: Physical component summary score
SD: Standard deviation
SF-36: Short Form 36 survey
VAS: Visual analogue scale
